# Vagus nerve stimulation improves coagulopathy in hemorrhagic shock: a thromboelastometric animal model study

**DOI:** 10.1186/1752-2897-8-15

**Published:** 2014-09-17

**Authors:** Joao B Rezende-Neto, Roger Lage Alves, Mario Carvalho, Thiago Almeida, Cyntia Trant, Christopher Kushmerick, Marcus Andrade, Sandro B Rizoli, Jose Cunha-Melo

**Affiliations:** 1 Department of General Surgery, St. Michael’s Hospital - University of Toronto, Toronto, ON, Canada; 2 Federal University of Minas Gerais, Belo Horizonte, Brazil

**Keywords:** Hemorrhage, Shock, Resuscitation, Trauma, Coagulopathy, Vagal nerve, Cytokines, Thromboelastometry

## Abstract

**Introduction:**

Inflammation plays a major role in the multifactorial process of trauma associated coagulopathy. The vagus nerve regulates the cholinergic anti-inflammatory pathway. We hypothesized that efferent vagus nerve stimulation (VNS) can improve coagulopathy by modulating the inflammatory response to hemorrhage.

**Methods:**

Wistar rats (n = 24) were divided in 3 groups: Group (G1) Sham hemorrhagic shock (HS); (G2) HS w/o VNS; (G3) HS followed by division of the vagus nerves and VNS of the distal stumps. Hemorrhage (45% of baseline MAPx15 minutes) was followed by normotensive resuscitation with LR. Vagus nerves were stimulated (3.5 mA, 5 Hz) for 30 sec 7 times. Samples were obtained at baseline and at 60 minutes for thromboelastometry (Rotem®) and cytokine assays (IL-1 and IL-10). ANOVA was used for statistical analysis; significance was set at *p* < 0.05.

**Results:**

Maximum clot firmness (MCF) significantly decreased in G2 after HS (71.5 ± 1.5 vs. 64 ± 1.6) (p < 0.05). MCF significantly increased in G3 compared to baseline (67.3 ± 2.7 vs. 71.5 ± 1.2) (p < 0.05). G3 also showed significant improvement in Alfa angle, and Clot Formation Time (CFT) compared to baseline. IL-1 increased significantly in group 2 and decrease in group 3, while IL-10 increased in group 3 (p < 0.05).

**Conclusions:**

Electrical stimulation of efferent vagus nerves, during resuscitation (G3), decreases inflammatory response to hemorrhage and improves coagulation.

## Introduction

Coagulopathy is linked to approximately 50% of the trauma associated deaths that occur within the first 48 hours after injury [1-3]. That condition is present in more than 25% of severely injured patients on admission, and is associated with a fourfold increased risk of death [1,4]. Severe trauma and hemorrhagic shock trigger major systemic inflammatory response. An exacerbated response leads to a pathophysiologic process that ultimately results in multiple organ failure [5-7]. There is a large body of evidence to support the correlation between excessive inflammation and coagulation dysfunction [7-12]. Therefore, inflammation is considered a key initiator of trauma associated coagulopathy [10,13,14].

The central nervous system modulates inflammatory response through hormonal and neuronal pathways. The vagus nerve has an important role in this process. Previous research demonstrated that electrical stimulation of the efferent vagus nerve inhibits pro-inflammatory cytokines through the “cholinergic anti-inflammatory pathway” [7,15-17]. Even though, previous studies have shown that vagus nerve stimulation protects against excessive inflammatory response in hemorrhagic shock, the effect on coagulation has been scarcely investigated [18]. Thus, the present study sought to investigate the acute hemostatic effect of VNS on coagulation and cytokine production in a controlled hemorrhagic shock model. The study was approved by the Animal Research Committee and of the Federal University of Minas Gerais, Brazil (protocol number 192/11).

## Methods

Male Wistar rats (200-250 g) were acclimated for 2 weeks, individually housed, and maintained at 25°C on 12-hour light/day cycles. Animals were fed rat chow (Ratochow-Purina; Caxias, Brazil), and water ad libitum.

### Experimental groups

Twenty-four (n = 24) animals were randomly divided in 3 groups (n = 8 per group) according to resuscitation regimen:

– Group 1 (Sham): Animals underwent surgical procedures but no hemorrhage.

– Group 2: Hemorrhage and resuscitation with lactated Ringer’s (LR) but no vagus nerve stimulation (VNS).

– Group 3: Hemorrhage followed by bilateral division of the vagus nerves and VNS of the distal stumps (efferent) during resuscitation.

### Pre-hemorrhage procedures and dissection of the vagus nerves

Animals were anesthetized with ketamine 60 mg/kg and xylazine 15 mg/kg (Fort Dodge Animal Health, Fort Dodge, IA) administered by intraperitoneal injection. Additional intravenous doses were given if needed. The right internal jugular vein and the right carotid artery were cannulated with 21G peripheral intravenous catheters previously filled with LR (Johnson & Johnson, Sao Jose dos Campos, Brazil). The vagus nerves were carefully dissected and tagged with a loop of 3–0 silk suture (Polysuture®, Sao Sebastiao do Paraiso, Minas Gerais, Brazil). Mean arterial pressure (MAP) was monitored continuously on the right carotid artery (Pro-Paq Protocol Systems, Beaverton, OR). A blood sample (1 ml) was obtained from the right carotid artery for baseline thromboelastometry (ROTEM® Coagulation Analyzer, Pentapharm, Munich, Germany). The sample was immediately transferred to a tube containing 3.2% sodium citrate as anticoagulant (MiniCollect®, Vacuette, Monroe, NC). Thromboelastometry was performed on temperature-corrected blood samples using NATEM® (Non-Activated TEM) and Star-tem® reagent to recalcify citrated blood (Ref. Number 503–01). Thromboelastometry parameters were calculated with the coagulation dynamics evaluation software (DyCoDerivAn; Avordusol, Rissov, Denmark). The following parameters were assessed: clotting time (CT), clot formation time (CFT), alpha angle (α), maximum clot firmness (MCF); at baseline and at the end of the experiment. A different blood sample was placed in a centrifuge tube (Eppendorf do Brasil Ltda, Sao Paulo, Brazil) with ethylenediaminetetra-acetic acid as anticoagulant for cytokine assay (IL-1β and IL-10). In brief, plasma was separated by centrifugation at 1600 × g for 15 minutes at 4°C. Samples were analyzed at a 1:5 dilution in potassium phosphate buffer. Enzyme-linked immunobsorbent assay plates (ELISA) (Neogen® Corporation, Indaiatuba, Brazil) were coated with sheep anti-rat IL-1β and IL-10 polyclonal antibodies (1–2 μg/ml) overnight. The plates were washed and blocked with 1% bovine serum albumin, and incubated overnight with samples of recombinant rat cytokine. Biotinylated polyclonal antibodies were used at a 1:1000 to 1:2000 dilution. The plates were washed and incubated with avidin-horseradish peroxidase (Dako North America Ltd., Carpinteria, CA) for 15 minutes. After a subsequent wash they were incubated with *o*-phenylenediamine and H_2_O_2_ for another 15 minutes. The reaction was stopped with sulfuric acid (150 μl/1.0 M); optical densities at 490 nm were determined. Cytokine assays were performed using blood samples obtained at baseline and at the end of the experiment.

### Hemorrhage/resuscitation procedures and vagus nerve stimulation

**Figure 1:**
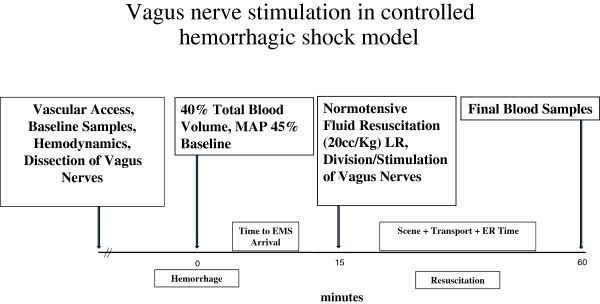
**Timeline of events and procedures during the experiment.** Hemorrhage was induced by withdrawal of 40% of total blood volume (TBV) and maintenance of mean arterial blood pressure (MAP) at 45% of baseline. LR = lactated Ringers.

Total blood volume was calculated as (TBV = 0.06 × Weight (g) + 0.77). Forty percent of the estimated TBV was taken from the right carotid artery. Thereafter, MAP was maintained at 45% of baseline level (MAP × 0.45) for 15 minutes (Figure 1). No fluids or blood products were given during this period to simulate Emergency Medical Services (EMS) arrival time. Resuscitation began with a bolus of LR (20 ml/Kg) infused over 3 minutes through the right internal jugular vein. Additional volumes of LR were infused to maintain MAP at baseline ± 5 mmHg. The resuscitation phase lasted for 45 minutes. Vagus nerves of group 3 animals were divided bilaterally. Subsequently, a total of seven stimuli were applied to the distal stump of the nerve bilaterally, during resuscitation, using a Grass Nerve Stimulator (Grass Technologies - Warwick, RI). Stimuli were 3.5 mA/5Hz and lasted for 30 seconds. A five minute interval was allowed between each stimulation (total time 35 minutes). Animals were euthanized with an anesthetic overdose after the final blood sample was obtained (Figure 1).

### Statistical analysis

One-way analysis of variance (ANOVA) was performed with post hoc analysis using Tukey’s test for multiple comparison between experimental means; data are reported as mean ± SEM, *p* < 0.05 was considered statistically significant.

## Results

### Hemodynamic response and cytokine levels

**Figure 2:**
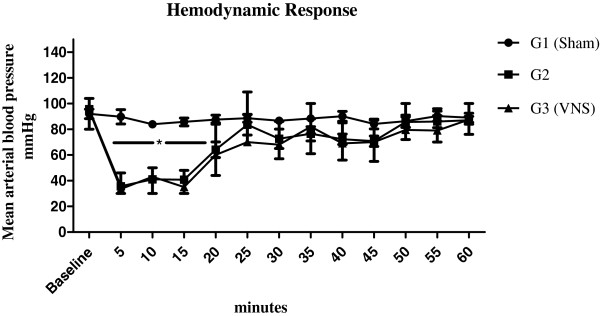
**Mean arterial blood pressure during hemorrhage and resuscitation.** Lactated Ringer’s infusion began at 15 minutes in groups 2 (no vagus nerve stimulation) and 3. Group 3 animals also underwent vagus nerve stimulation starting at the 15^th^ minute. Data represent mean ± SEM (8 animals per group). ^*^p < 0.05 vs. baseline and sham; one way analysis of variance (ANOVA). VNS = Vagus nerve stimulation, G = group.

Average MAP was 91.1 ± 1.8 mmHg at baseline among the groups (*p* > 0.05). Bleeding provoked a significant decrease in MAP in groups 2 and 3 compared to sham (G1), and this difference persisted throughout the first 20 minutes of the experiment; respectively 62.3 ± 3.7 mmHg vs. 85.5 ± 3.5 mmHg (*p* < 0.05). Mean arterial pressure response to bleeding and resuscitation was unaffected by VNS (group 3) compared to group 2; *p* > 0.05 (Figure 2). There was no significant difference in the volumes of lactated Ringer’s used during resuscitation between groups 2 and 3, respectively 4.8 ± 0.3 ml and 4.6 ± 0.2 ml (*p* > 0.05).

**Figure 3:**
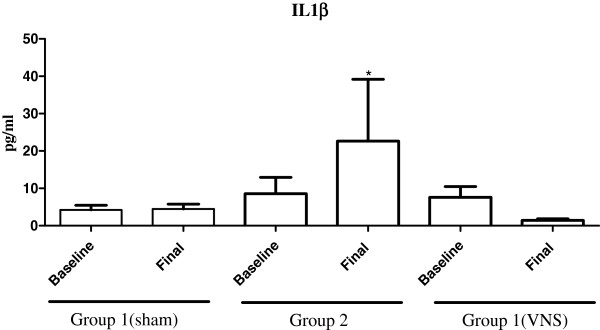
**Interleukin 1β plasma levels.** Samples obtained at baseline and after resuscitation. Data represent mean ± SEM (8 animals per group). ^*^p < 0.05 vs. baseline group 2 (resuscitation without vagus nerve stimulation), final group 1 (sham), and final group 3 (resuscitation with vagus nerve stimulation); one way analysis of variance (ANOVA). VNS = Vagus nerve stimulation.

Results showed that group 2 animals had a significant increase in plasma IL-1 after hemorrhage/resuscitation compared to baseline and final sham; respectively 8.5 ± 4.3 pg/ml vs. 22.6 ± 10.5 pg/ml, and 4.5 ± 1.3 vs. 22.6 ± 10.5 pg/ml (*p* < 0.05). In contrast, IL-1 levels decreased significantly when VNS was performed during resuscitation (group 3) compared to baseline and final group 2; respectively 7.7 ± 2.8 vs. 0.7 ± 0.5 and 22.6 ± 10.5 vs. 0.7 ± 0.5 pg/ml (*p* < 0.05). As expected, sham hemorrhage did not alter IL-1 levels significantly (Figure 3). Group 3 animals were also assessed for anti-inflammatory cytokine IL-10. There was a significant increase in plasma IL-10 levels with VNS during hemorrhage resuscitation compared to baseline; respectively 26.3 ± 3.5 pg/ml vs. 40.2 ± 6.0 pg/ml (*p* < 0.05).

### Thromboelastometric parameters

**Figure 4:**
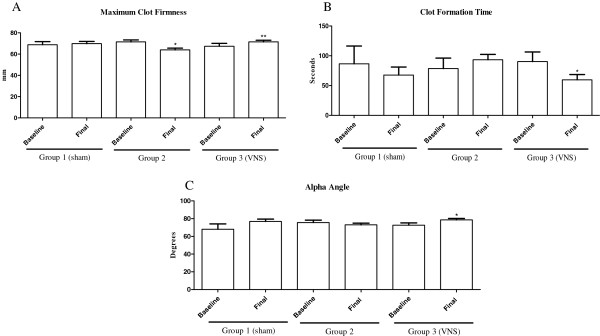
**Rotational thromboelastometry.** Samples obtained at baseline and after resuscitation. Data represent mean ± SEM (8 animals per group). **A**, ^*^p < 0.05 vs. baseline group 2 (resuscitation without vagus nerve stimulation); ^**^p < 0.05 vs. baseline group 3 (vagus nerve stimulation). **B**, ^*^p < 0.05 vs. baseline group 3. **C**, ^*^p < 0.05 vs. baseline group 3. VNS = Vagus nerve stimulation.

Hemorrhage and resuscitation without VNS (group 2) resulted in a significant decrease in MCF compared to baseline; respectively 71.5 ± 1.8 mm vs. 64.0 ± 1.7 mm (*p* < 0.05). In contrast, when VNS was performed during resuscitation (group 3) MCF increased significantly compared to baseline 67.3 ± 2.7 mm vs. 74.5 ± 1.5 mm (*p* < 0.05) (Figure 4-A). Vagus nerve stimulation during resuscitation also improved other tromboelastometric parameters in group 3. The alfa angle increased significantly 72.7 ± 2.5^o^ vs. 78.7 ± 1.5° (*p* < 0.05), while the CFT decreased 90.3 ± 16.2 vs. 59.7 ± 8.9 seconds (*p* < 0.05); compared to baseline. Hemorrhage followed by resuscitation without VNS (group 2) did not improve those parameters (Figure 4-B and C).

## Discussion

Our results indicate that efferent VNS, during hemorrhagic shock resuscitation, improves hemostasis by decreasing inflammatory response. Immunomodulatory effects of efferent VNS result from the interaction between acetylcholine and the nicotinic receptor α7 subunit in immune cells, this process is denominated “cholinergic anti-inflammatory pathway” [15,16]. Stimulation of this receptor’s subunit also inhibits the activation of nuclear factor (NF-κB), high mobility group B1 (HMGB1), IL-1β, TNFα, and other pro-inflammatory cytokines [15-17,19]. Immunomodulation induced by VNS was previously described in a wide range of experimental models particularly those involving septic challenges [15,20-22].

Cytokines play a major role in the systemic inflammatory response unleashed by severe trauma, and this condition promotes abnormal hemostasis [23-25]. Inflammatory dysfunction of endothelial cells is an important event in this process. Accordingly, previous research showed that shedding of syndecan-1, a proteoglycan component of endothelial cell glycocalyx, facilitates resolution of inflammation and clearance of chemokines [26]. In keeping with that hypothesis, data from hemorrhagic shock trauma patients showed that interferon-γ, fractalkine, and IL1-β correlated negatively with syndecan-1 shedding, whereas IL-10 had a positive correlation [27]. Interestingly, our findings showed an increase in plasma IL-10 levels and reduction in IL-1β in response to VNS. Because inflammation can produce both inhibition and exacerbation of the clotting process it is conceivable to hypothesize that VNS produced a more balanced hemostatic response that ultimately resulted in improved coagulation; evidenced in the present study by improved clot formation kinetics and enhanced clot firmness. Moreover, previous research showed that IL-10 not only decreases NF-κB activation, expression of intracellular adhesion molecule 1 (ICAM-1), and vascular cell adhesion molecule 1 (VCAM-1), but also inhibits pro-inflammatory cytokine production in a feedback loop [28,29]. Thereby protecting endothelial cells from injury provoked by excessive inflammatory response. Accordingly, higher levels of IL-10 and decreased levels of IL-1β, after VNS in our model, could have decreased hemorrhage induced endothelial dysfunction and also contributed to better hemostasis.

Our findings provide additional information compared to a recent study wherein the hemostatic effect of VNS was investigated by means of a peripheral hemorrhage animal model [18]. In our study, hemorrhage was more severe and resulted in significant hypotension setting the stage for major inflammatory response. Thus, we believe that this model was more suitable to investigate the immunomodulatory effect of VNS on hemostasis. Our results also corroborate previous research that showed no cardiovascular dysfunction as a result of efferent VNS [30-33]. Evidenced by similar hemodynamic response and fluid requirements, despite severe hemorrhage and regardless of VNS. Several different strategies of VNS are reported in the literature [15,16,18,20-22,34]. Using the same hemorrhage model described herein, we performed pilot experiments to determine what would be the best method for this study. Interestingly, we found no difference in the coagulation profile or in cytokine levels when vagus nerves were stimulated but not divided (data not shown). Likewise, bilateral vagotomy only or stimulation of the proximal stumps after vagotomy did not result in significant improvement in coagulation or change in cytokine levels compared to non-stimulated vagus (data not shown).

This study has several limitations. Inter-species comparison showed that rats are particularly hypercoagulable [35]. This could potentially have augmented the procoagulant effect provoked by VNS. Furthermore, we did assess for fibrinolysis, which was recently described as an independent predictor of mortality in trauma patients [36,37]. Even though significant hypotension was produced in the animals, we did not simulate major tissue injury or assess the degree of hypoperfusion. Moreover, blood products were not used during resuscitation restricting the clinical relevance of the model.

## Conclusions

This study demonstrated that efferent VNS during hemorrhagic resuscitation improves coagulation. Even though the precise mechanism was not investigated, activation of the cholinergic anti-inflammatory pathway was involved in that response.

## Competing interests

The authors declare that they have no competing interests.

## Authors’ contributions

JBRN, conceived the study, participated in the design and coordination, drafted the manuscript and performed statistical analysis. RLA, participated in the design and manuscript drafting, performed the assays and surgical procedures. MC Jr., Participated in the design of the study and performed the assays and surgical procedures. TA, Participated in the design of the study, performed the assays and surgical procedures. CT, Performed the assays and surgical procedures, participated in the manuscript drafting. CK, participated in the design of the study and surgical procedures. MA, Performed the assays, statistical analysis and manuscript drafting. SBR, participated in the design, coordination, and manuscript drafting. JRCM participated in the design, coordination, and performed the assays. All authors read and approved the final manuscript.
